# Exploring how leisure-time physical activity moderate the school bullying–trauma network among children: the role of body image

**DOI:** 10.3389/fpsyg.2026.1804968

**Published:** 2026-08-19

**Authors:** Junying Liu, Yonghui Wang, Yifan Zhang, Haiting Zhu

**Affiliations:** 1 Faculty of Education, Shaanxi Normal University, Xi'an, China; 2 Zhangjiazhuang Branch of Kunlun Primary School in Xincheng District, Xi'an, China; 3 School of Physical Education, Shaanxi Normal University, Xi'an, China; 4 Department of Tourism, University of Otago, Dunedin, New Zealand

**Keywords:** body appreciation, children, leisure-time physical activity, network analysis, PTSD, school bullying victimization

## Abstract

**Background:**

Bullying is a prevalent form of interpersonal aggression that can trigger post-traumatic stress disorder (PTSD) symptoms in children. However, the interplay between different forms of school bullying, PTSD symptom clusters, and body-related factors remains unclear. Body image, defined as the perceptions and attitudes toward body, is closely tied to the emotional wellbeing and sensitivity to peer experiences, making it an important factor in understanding the psychological impact of bullying. Leisure-time physical activity, known to be associated with body perceptions, thereby may also shape the response to bullying victimization.

**Methods:**

A total of 1,686 Chinese primary school students (M age = 10.95, SD = 0.85, 53.6% male) completed self-report measures of bullying victimization, PTSD symptoms, body appreciation, and physical activity. The Gaussian Graphical Model (GGM) was used, with the Bridge Expected Influence (BEI) as an index reflecting the extent of connecting distinct communities within the network. The moderating effect of leisure-time physical activity was examined using the Moderated Network Model (MNM).

**Results:**

Verbal bullying and alienation emerged as primary bridge nodes (i.e., variables that connect victimization and PTSD symptom communities in the network), while body appreciation is negatively associated with verbal bullying and alienation. MNM showed that leisure-time physical activity moderated the relationship between body appreciation and alienation as well as the link between body appreciation and verbal bullying.

**Conclusion:**

Body appreciation is a crucial psychological resource buffering bullying-related trauma. In addition, findings also support leisure-time physical activity as a valuable opportunity for self-expression at the social level, which may contribute to the recovery from bullying-related trauma.

## 1 Introduction

Bullying can be defined as a long-lasting and systematic form of interpersonal aggression where an individual is persistently and over time exposed to negative actions, and where the target finds it difficult to defend her−/himself against these actions (Olweus, 2013). These negative actions include, but are not limited to, verbal hostility, inappropriate comments, aggressive behaviour, physical violence and social exclusion. The problem has become even worse with the increasing prevalence of cyberbullying.

Children, in particular, are highly vulnerable to bullying. This developmental stage is marked by rapid psychological and social changes, including identity formation, peer dependence, and a strong need for social belonging. These characteristics may amplify the adverse effects of bullying and render children especially vulnerable to its harmful consequences. A recent meta-analysis involving more than 600,000 participants estimated that approximately one in four children (25%; 95% CI: 22–28%) experience bullying victimization (Ariani et al., 2025). Another large-scale school bullying survey involving nearly 100,000 Chinese participants found that 68,342 students (71.6%) experienced school bullying of varying types and degrees. Among them, 56,372 students experienced mild bullying and 11,970 students experienced severe bullying, accounting for 59 and 12.5% of the total sample, respectively (Zhao et al., 2024).

Bullying is a form of aggression characterized by maltreatment or abuse and has therefore been recognized as a potentially traumatic experience (Kaess, 2018). Empirical evidence indicates that exposure to bullying, whether in school or workplace settings, constitutes a strong risk factor for the development of posttraumatic stress disorder (PTSD) (Idsoe et al., 2012; Nielsen et al., 2015; Spence Laschinger and Nosko, 2015). Research on childhood bullying has shown that repeated exposure to peer victimization can lead to trauma responses similar to those seen in abuse survivors (Ossa et al., 2019). For example, a cross-sectional study found that approximately 27.6–40.5% of bullied students met the clinical criteria for PTSD symptoms, including intrusive thoughts and avoidance (Idsoe et al., 2012).

### 1.1 Established relationship between school bullying and PTSD

PTSD is a mental disorder that may develop after an individual experiences or witnesses a traumatic event. When a person is exposed to severe trauma and subsequently exhibits specific patterns of symptoms in response, the disorder can be diagnosed as PTSD (American Psychiatric Association, 2013). While trauma is explicitly defined in diagnostic manuals as death, threatened death, actual or threatened serious injury, or actual or threatened sexual violence, PTSD may also develop as a result of the cumulative impact of numerous minor, non–life-threatening stressors (Cloitre, 2021). Bullying exemplifies this form of trauma, typically involving exposure to non-life physical harm but also diverse forms of non-physical assaults, including verbal bullying. Prolonged exposure to such experiences can profoundly disrupt core cognitive schemas regarding the self, others, and the external world (Mikkelsen and Einarsen, 2002). Abrupt alterations in these fundamental structures may be perceived as severely threatening and thereby give rise to traumatic responses (Janoff-Bulman, 1992). Research has shown that traumatic events rooted in interpersonal violence are more likely to precipitate PTSD than those arising from natural disasters (Liu et al., 2017). A meta-study of 29 studies on bullying further revealed that 57% of victims scored above the diagnostic threshold for PTSD (Ossa et al., 2019).

Studies have demonstrated that the core symptom clusters of PTSD can be conceptualized within both four-factor and five-factor models (Asmundson et al., 2000; Elhai and Palmieri, 2011). The Diagnostic and Statistical Manual of Mental Disorders, Fifth Edition (DSM-5) formally adopts a four-factor model, which organizes symptoms into intrusion, avoidance, negative alterations in cognition and mood, and alterations in arousal and reactivity (American Psychiatric Association, 2013). The core symptomatology of PTSD in children and adolescents largely mirrors that of adults (Greenwald and Rubin, 1999).

There are three common forms of bullying victimization: verbal, involving harmful language such as teasing or threats; social, which undermines children’ relationships through exclusion or rumors; and physical, entailing direct harm to the body or belongings (Bear et al., 2011). With the rapid development of digital technologies and online communication, cyberbullying has become increasingly prevalent. Cyberbullying is a form of bullying that is conducted through modern information and communication technology (Smith, 2012). Together, these forms demonstrate the complex nature of bullying and its detrimental effects on children’ wellbeing.

Studies have consistently shown that verbal aggression is the most common form of bullying in school settings, occurring more frequently than physical aggression (Fernández-Aldana et al., 2024; Poling et al., 2019; Wang et al., 2009). Verbal harassment, including insults, threats, intimidation, and discrediting, appears especially widespread. Using Latent Profile Analysis, one study examined the co-occurrence of three forms of bullying victimization among Chinese children. The findings indicated that verbal bullying was the most prevalent form, yet it frequently co-occurred with physical and social bullying and was associated with psychological distress, including depression and anxiety (Wei et al., 2019). In addition, appearance-related cyberbullying can be conceptualized as a form of verbal aggression enacted in online contexts, involving negative comments or ridicule targeting individuals’ physical appearance (Berne et al., 2014).

While different forms of bullying are interrelated and often co-occur, each form may have distinct psychological consequences. Although prior studies have documented that bullying victimization is associated with elevated PTSD symptoms (Idsoe et al., 2020; Li et al., 2023), the specific links between types of bullying and individual PTSD symptom clusters remain underexplored. Investigating these associations is critical, as certain forms of bullying may act as risk factors that differentially influence core PTSD symptoms, including intrusion, alienation, and somatic symptoms.

To address this complexity, network analysis provides a methodological approach. Unlike traditional models that treat bullying or PTSD as unitary constructs, network analysis can conceptualize individual bullying experiences as potential risk factors directly connected to specific PTSD symptoms. This method allows the identification of core symptoms that play a central role in the symptom network and bridge pathways through which particular forms of bullying may trigger or maintain PTSD symptoms. Therefore, the primary aim of the present study is to explore whether different forms of bullying exhibit distinct associations with specific PTSD symptoms, using network analysis to identify bridge factors/symptoms within these relationships.

### 1.2 Positive body image as a protective psychological resource

Children whose body types deviate from socially prescribed ideals are often subjected to bullying behaviors, with peers ridiculing or mocking them by highlighting such physical differences (Pearce et al., 2002). Importantly, the notion of “body type” in this context extends beyond objective indicators such as weight and body size, encompassing also the subjective perception of one’s weight and image toward one’s body. Research has demonstrated that physical esteem and body image dissatisfaction mediate the positive association between actual weight and experiences of bullying victimization (Brixval et al., 2012; Fox and Farrow, 2009). Bullying victimization risk is heightened by both actual overweight and the subjective perception of overweight (Midei and Matthews, 2011; Sentenac et al., 2012).

Unlike positive body image, which is linked to resilience and wellbeing, negative body image is often accompanied by body dissatisfaction, diminished physical esteem, and related psychological issues. Children with a negative body image have been found to be substantially more likely to experience bullying victimization (Holubcikova et al., 2015). Moreover, low self-evaluation and self-esteem have also been associated with heightened susceptibility to bullying (Raskauskas et al., 2015). Complementary evidence suggests that students with lower academic self-evaluations are particularly vulnerable to such victimization (Thijs and Verkuyten, 2008). These psychological characteristics are closely aligned with the broader construct of negative body image.

By contrast, positive body image, characterized by appreciation, respect, and acceptance of one’s body, has been identified as a salient protective factor in child mental health (Tylka and Wood-Barcalow, 2015b). Studies have shown that children with a positive body image are more likely to exhibit higher self-esteem and lower levels of depression and anxiety (Linardon et al., 2022; Tort-Nasarre et al., 2023). Importantly, emotion regulation and resilience can serve as critical buffers against the adverse effects of bullying. Thus, such protective factors may alleviate the psychological distress associated with bullying victimization, particularly in the development of PTSD symptoms.

Taken together, these findings underscore the importance of body image as a risk and/or protective factor in child mental health. Accordingly, the second aim of the present study was to examine how body image is embedded within the network structure linking bullying victimization and PTSD symptoms, with particular attention to whether body image functions as a bridge factor connecting victimization experiences and symptomatology.

### 1.3 The role of leisure-time physical activity

Life is filled with a wide range of activities, both pleasant and unpleasant, which are commonly categorised into leisure, work, and nonwork obligations (Stebbins, 2012). Within this framework, sport can be recognized as one form of leisure-time physical activity, while planned exercise represents another, both contributing to health benefits (Berg et al., 2015). However, the intended outcomes of them diverge. Planned exercise (e.g., aerobics, strength training) emphasizes individual and self-directed goals, such as increasing personal health, fitness, and wellbeing. In contrast, sport (e.g., football, basketball) emphasizes social interaction, competition, and collective participation, with primary objectives centered on skill development, teamwork, enjoyment, and achieving success within a set of rules (Fraser-Thomas et al., 2005). Despite these differences, both forms contribute to overall physical activity, which has been widely recognized as essential for healthy development during childhood and adolescence.

The importance of promoting physical activity during childhood and adolescence has also been emphasized from a public health perspective. The World Health Organization (WHO) recommends that children and adolescents accumulate an average of at least 60 min of moderate- to vigorous-intensity physical activity per day across the week, with vigorous-intensity and muscle- and bone-strengthening activities incorporated at least 3 days per week (Bull et al., 2020). These recommendations reflect the well-established role of physical activity in promoting physical, psychological, and social health among young people. Although physical activity can be accumulated across multiple domains, leisure-time physical activity is particularly relevant to the present study because it represents a voluntary and self-directed context in which health, psychological wellbeing, and social development may be promoted.

Certainly, leisure-time physical activity can be understood as more than a form of bodily movement. It represents a meaningful domain of human experience associated with freedom, creativity, and self-realization (Stebbins, 2015). Rather than being physical activity alone, such practices involve experiential, emotional, and symbolic dimensions through which individuals engage with their sense of self (Pieper, 2009). When a person engages in running (Zhu et al., 2026), swimming (Wheaton, 2010), or yoga (Liu et al., 2022), the experience may function as an expression of agency and competence, reinforcing perceptions of autonomy and personal capability (Deci and Ryan, 2008). This sense of agency and accomplishment contributes to more enduring forms of wellbeing compared to passive or consumption-based leisure (Csikszentmihalyi, 2009; Stebbins, 2015), aligning with broader psychological frameworks of eudaimonic wellbeing.

For individuals experiencing psychological distress, such as PTSD or the effects of bullying, leisure-time physical activity may provide an alternative space for identity reconstruction and emotional regulation. Physical activity has been shown to reduce symptoms of stress and trauma while simultaneously supporting the development of self-efficacy and resilience (Rosenbaum et al., 2015). For those experiencing bullying, participation in leisure and sport activities can foster social connection, belonging, and a renewed sense of self, thereby counteracting experiences of isolation and marginalization (Jansen et al., 2014). In this sense, leisure-time physical activity can be understood not merely as a distraction from adversity, but as a socially and psychologically meaningful practice that supports processes of recovery, identity formation, and wellbeing.

In line with the WHO recommendations, which recognize sports, active recreation, and planned exercise as important domains through which children and adolescents can accumulate health-enhancing physical activity, the present study did not distinguish between sport and planned exercise. Instead, they were conceptualized as a single category of leisure-time physical activity after school (hereinafter referred to as “physical activity”). Accordingly, this study further hypothesized that the network structure of bullying victimization in relation to PTSD symptoms varies as a function of physical activity level, with distinct network characteristics expected in high versus low level. To test this hypothesis, we examined whether physical activity moderated the network structure between bullying victimization and PTSD symptoms.

In general terms, the Moderated Network Model (MNM) allows us to examine whether and how the relationships between variables in a network change depending on the level of a moderator—in this case, physical activity (Haslbeck et al., 2021). This approach provides a more nuanced understanding of whether physical activity may alter the structure of the network linking bullying victimization and PTSD symptoms, rather than simply comparing networks between high and low physical activity groups. By contrast, the Network Comparison Test (NCT), which is commonly used for group comparisons, may be unstable in small or unbalanced samples (Haslbeck, 2022). Thus, the MNM, which incorporates physical activity directly as a moderator, provides a more flexible and robust framework for detecting such network differences.

## 2 Methods

### 2.1 Participants and procedure

A cluster-based sampling approach was employed in this study. In May 2025, with approval from the principals of two primary schools in Xi’an, 34 classes across Grades 4 to 6 were included. Eligible participants were students who were able to understand and complete the questionnaires independently and who provided written informed consent from both themselves and their parents or legal guardians. Of the 1,732 students who participated, 1,686 were included in the final analyses after excluding questionnaires with substantial missing data or invalid response patterns, resulting in a response rate of 97.3%. The mean age of participants was 10.95 years (SD = 0.85).

The study protocol was approved by the Shaanxi Normal University Human Ethics Committee (Approval No. 2025-106). All procedures involving human participants were conducted in accordance with the ethical standards of the institutional review board. The questionnaires were administered by trained research assistants in classroom settings following standardized procedures. Before administration, participants received standardized instructions, and trained research personnel were available to answer procedural questions without influencing participants’ responses. Classroom teachers were present only to maintain classroom order and did not participate in questionnaire administration. Participants completed the questionnaires independently within approximately 25 min.

### 2.2 Measures

#### 2.2.1 Delaware bullying victimization scale–student form

This study employed the Chinese version of the Delaware Bullying Victimization Scale–Student Form (DBVS–S) (Bear et al., 2011; Xie et al., 2015; Xie et al., 2018). As the present study focuses on school-based bullying and the construct of school climate, the cyberbullying dimension was excluded to maintain conceptual consistency and ensure that the measurement captured behaviors occurring primarily within the school context. The final version used in this study, therefore, comprised 12 items across three dimensions: verbal bullying (4 items), relational bullying (4 items), and physical bullying (4 items). Psychometric properties of all measures in their Chinese versions, including internal consistency and test–retest reliability, are provided in the Supplementary materials.

#### 2.2.2 Child report of post-traumatic symptoms

The Chinese version of the Child Report of Post-Traumatic Symptoms (CROPS) was used to assess post-traumatic symptoms in children aged 7–17, with higher scores indicating greater symptom severity (Greenwald and Rubin, 1999; Liao, 2007). Although the original CROPS was developed as a unidimensional scale, subsequent research has supported a three-factor structure, comprising Intrusion, Somatic Symptoms, and Alienation (Edner et al., 2017). The hypothesized three-factor model was generally supported in the present sample of Chinese children by using confirmatory factor analysis. The fit indices (CFI = 0.806, RMSEA = 0.077, and SRMR = 0.069) indicated that the model fit was within an acceptable range, although not optimal.

#### 2.2.3 Body image-related variables

Regarding body image-related dimensions, we first evaluated positive body image using the Chinese version of the Body Appreciation Scale-2 (BAS-2). This scale provides a brief self-report measure designed to assess individuals’ acceptance, respect, and favorable attitudes toward their bodies (Tylka and Wood-Barcalow, 2015a; Wu et al., 2024).

In addition, overweight preoccupation was assessed to capture the subjective evaluation of fat anxiety and weight vigilance. This measure was derived from the Chinese version of the Multidimensional Body–Self Relations Questionnaire (MBSRQ), with higher scores on the overweight preoccupation subscale indicating greater concern or anxiety about body weight (Brown et al., 1990; Ma, 2006).

#### 2.2.4 Physical activity rating scale

The Chinese version of the Physical Activity Rating Scale (PARS-3) was used in this study (Liang, 1994). The PARS-3 assessed leisure-time physical activity through three dimensions: frequency, duration per session, and intensity, each rated on a five-point Likert scale. For example, one of the items asked about the frequency of physical activity/games after school.

The composite score was calculated as the product of frequency, duration, and intensity, following the original PARS-3 scoring procedure (Liang, 1994). Because this multiplicative scoring method may produce a positively skewed distribution, the composite score was natural log (LN)-transformed before inferential analyses to improve model assumptions, reduce the influence of extreme values, and mitigate potential collinearity associated with multiplicative terms. The transformation was used solely for statistical estimation; higher transformed scores still indicate higher overall levels of leisure-time physical activity.

### 2.3 Statistical analysis

#### 2.3.1 Gaussian graphical model

A Gaussian Graphical Model (GGM) with EBICglasso regularization was first estimated using the R package *qgraph* to construct a baseline cross-sectional network of PTSD symptoms, bullying victimization subscales and variables related to body image. The EBICglasso procedure with LASSO regularization (*γ* = 0.5) was applied to shrink spurious small edge estimates toward zero, yielding a sparse and interpretable network structure (Haslbeck and Waldorp, 2018). This unconditional network allowed us to identify the core structure and direct associations among variables, providing a benchmark against which moderation effects could later be evaluated through MNM.

Furthermore, Bridge Expected Influence (BEI) was calculated from edge weights in both absolute and signed forms, allowing for the identification of variables that served as key bridges linking the symptom and risk-factor communities while distinguishing the overall strength (absolute BEI) from the directional influence (signed BEI). Specifically, absolute BEI reflects the overall strength of a node’s connections to nodes in other communities, regardless of direction, with larger values indicating stronger bridging effects. In contrast, signed BEI retains the direction of associations: positive values indicate that a node is primarily connected to other communities through positive relationships, whereas negative values indicate predominantly negative relationships. As there are no universally established cutoff thresholds for statistical “significance” for BEI, these values are interpreted comparatively within the network, with particular attention given to nodes exhibiting relatively larger absolute BEI (Jones et al., 2021).

The stability of the baseline cross-sectional network was evaluated using non-parametric bootstrap procedures implemented in the R package *bootnet*. Edge-weight accuracy was assessed by generating 1,000 bootstrap samples to estimate 95% confidence intervals. Additionally, case-dropping bootstrap was performed to examine the stability of BEI values, quantified by the correlation stability coefficient (CS-coefficient) (Epskamp et al., 2018).

#### 2.3.2 Moderated network model

Following the establishment and stability assessment of the baseline network, MNM was conducted using the R package mgm to investigate whether physical activity moderated the associations among PTSD symptoms (Haslbeck et al., 2021), bullying victimization subscales, and variables related to body image. LASSO regularization was applied, with the regularization parameter selected via cross-validation (CV), a widely used method for identifying the optimal LASSO parameter (Chetverikov et al., 2021). And the ruleReg argument was set to “AND” to provide conservative estimates of pairwise and interaction effects, minimizing overestimation of edges. In the MNM, pairwise interactions represent the main associations between nodes, while three-way interactions represent the moderating effect of physical activity on these associations.

The stability of the network and its estimated parameters was evaluated using 1,000 non-parametric bootstrap samples via the resample function, generating empirical sampling distributions and 95% confidence intervals. Low variance in these distributions indicated a stable network structure. Three-way interactions whose confidence intervals did not cross zero were interpreted as a moderating effect of physical activity on the associations between nodes.

## 3 Results

### 3.1 Demographic characteristics

The study sample consisted of 1,686 children aged 10 to 13 years (*M* = 10.95, SD = 0.85), including 904 boys (53.6%) and 782 girls (46.4%). Regarding the frequency of bullying victimization, 413 children (24.5%) reported never experiencing bullying. Among those who had experienced bullying, 936 (55.5%) reported a frequency below “occasionally” (scores 1 to 2), 236 (14.0%) reported occasional bullying occurring less than twice per month (scores 2 to 3), and 101 (6.0%) reported being bullied more than twice per month (scores above 3). 75.5% of children engaged in low, 16.2% in moderate, and 8.3% in high physical activity levels, according to the standards of the Physical Activity Rating Scale (see Table 1).

**Table 1:** Characteristics of the study population and study variables (*N* = 1,686).

Characteristic	Variable	Domain	Value
Study population	Age		10.95 ± 0.85
	BMI		28.92 ± 11.45
	Sex, *n* (%)		
	Boys		904 (53.6%)
	Girls		782 (46.4%)
Study variables	Verbal bullying	Bullying	1.93 ± 1.11
	Physical bullying	Bullying	1.36 ± 0.69
	Relational bullying	Bullying	1.40 ± 0.76
	Alienation	PTSD	0.37 ± 0.42
	Intrusion	PTSD	0.61 ± 0.42
	Somatic symptoms	PTSD	0.39 ± 0.47
	Overweight preoccupation	Body image	2.50 ± 1.00
	Body appreciation	Body image	5.98 ± 1.11
	Physical activity (PARS-3 Original)	Moderator	40.81 ± 18.70

Unless otherwise specified, continuous variables are presented as mean ± SD, and categorical variables are presented as *n* (%).

### 3.2 Baseline GGM results

**Figure 1:**
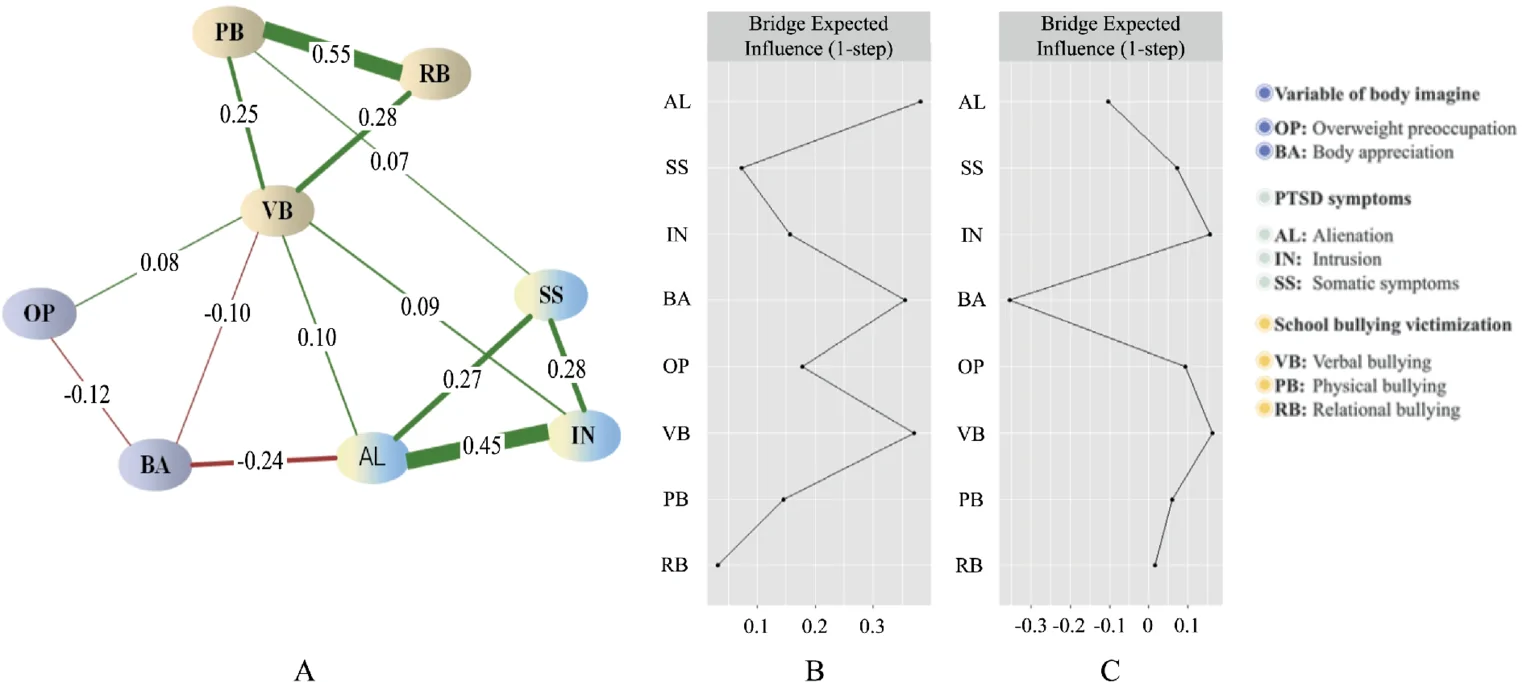
Baseline network of PTSD symptoms, bullying victimization and variables related to body image. **(A)** The green edges represent positive relations and the red edges represent negative relations. The figures on the lines represent edge weights between nodes. **(B)** Absolute BEI (*Z* score) of the network. **(C)** Signed BEI (Z score) of the network.

Figure 1A illustrates the network structure of bullying victimization and PTSD. Within the symptom cluster, alienation exhibited the strongest cross-community strength (absolute BEI = 0.38, signed BEI = −0.10), whereas verbal bullying demonstrated the strongest cross-community strength within the victimization cluster (absolute BEI = 0.37, signed BEI = −0.16). In addition, body appreciation bridged the symptom and victimization clusters in a buffering manner, with an absolute BEI of 0.35 and a signed BEI of −0.35 (As shown in Figures 1B,C).

The stability of the baseline cross-sectional network was supported by the bootstrap analyses. Non-parametric bootstrap procedures indicated that the majority of edge weights were estimated with high precision, with 95% confidence intervals generally narrow and not overlapping zero. This suggests that the observed network connections are robust (Supplementary Figure S1). Furthermore, case-dropping bootstrap analyses demonstrated that bridge expected influence (BEI) values were stable, with a CS-coefficient of 0.672 (Supplementary Figure S1).

### 3.3 MNM results

**Figure 2:**
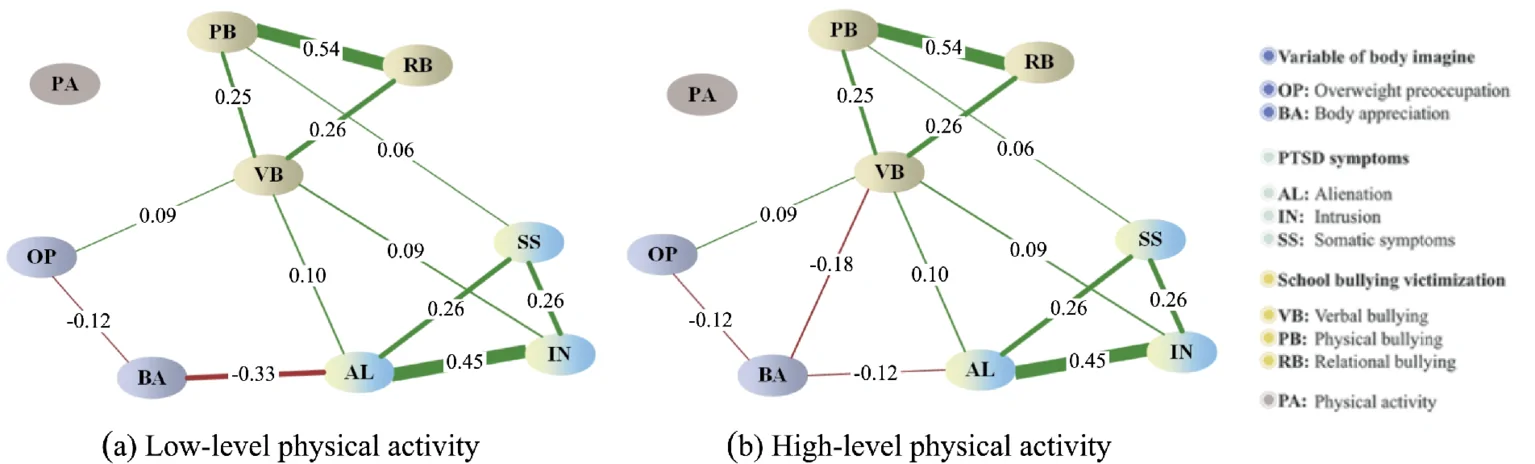
Moderated network structure with physical activity a moderator. The green edges represent positive relations, whereas the red edges represent negative relations. As physical activity serves as the moderator conditioned in the network, no edges are connected to this variable; it is displayed in the visualization only to indicate its moderating role. Panels **(a,b)** depict the network structures at low and high levels of physical activity, respectively. These levels correspond to values one standard deviation below and above the mean.

The MMN results revealed one positive and one negative three-way interaction (As shown in Figure 2). The moderating effects of physical activity emerged within the two-way interactions of body appreciation with alienation and body appreciation with verbal bullying. Specifically, physical activity attenuated the negative association between body appreciation and alienation (effect = 0.11), indicating that as physical activity increased, this relationship became less strongly negative. In contrast, the two-way interaction between body appreciation and verbal bullying intensified with higher levels of physical activity (effect = −0.09). At low levels of physical activity (one standard deviation below the mean), no association was observed between body appreciation and verbal bullying; however, the magnitude of the negative association increased progressively as physical activity rose.

The nonparametric bootstrapped sampling distributions of the MNM are presented in Supplementary Figure S2. The relatively narrow distributions observed for two-way interactions (pairwise effects) suggested good stability of the edge weight estimates. In comparison, the distributions for three-way interactions (moderation effects) showed greater variability. Within these, the moderation effect of physical activity on body appreciation and alienation was somewhat stable. However, the moderation effect on body appreciation and verbal bullying appeared limited stability. These findings should therefore be interpreted with caution, particularly regarding the latter one.

## 4 Discussion

### 4.1 Bridging variables linking bullying victimization, body image, and PTSD symptoms

The present study first examined the network structure linking bullying victimization, body image-related variables, and PTSD symptoms among children. In particular, we aimed to identify potential bridging variables that connect these domains. In network analysis, three key bridging variables were identified: alienation, verbal bullying and body appreciation. Within the symptom cluster, alienation (including social isolation, negative self-appraisal, negative affect and pessimism about the future) exhibited the strongest cross-community connectivity, suggesting it may serve as a central pathway through which bullying experiences and body image relate to PTSD symptoms.

This finding is consistent with previous research showing that loneliness and other cognitive factors mediate the relationship between bullying victimization and PTSD (Li et al., 2023). These findings should also be interpreted within the developmental context of childhood. During this period, cognitive appraisal, emotional regulation, and self-concept are still developing, making children more susceptible to interpersonal experiences such as bullying. Consequently, negative peer interactions may be more readily internalized into self-evaluations and emotional responses, increasing vulnerability to post-traumatic symptoms compared with more mature developmental stages. Bullying victimization can engender enduring fear and insecurity (Bell et al., 2019). Such insecurity may further increase children’s susceptibility to negative self-appraisals (including, but not limited to, evaluations of their body) as well as negative perceptions of others (Zhu and Zhang, 2025). These maladaptive cognitive patterns may undermine interpersonal trust, thereby limiting access to the comfort and benefits derived from social support. The resulting social withdrawal can intensify feelings of loneliness, which, in turn, may further exacerbate PTSD symptoms.

Among the victimization cluster, verbal bullying demonstrated the highest cross-community bridging strength, indicating its central role in linking the risk and/or protective factor community with the PTSD symptom community within the network. Verbal bullying is not only the most prevalent form of school bullying but is also positively associated with the onset of PTSD (Poling et al., 2019). Prior research has shown that verbal bullying, particularly ridicule and insults targeting physical appearance, is closely related to negative body image (Day et al., 2021). Such experiences may heighten body dissatisfaction, thereby increasing vulnerability to eating disorders, PTSD, and other psychopathologies (Day et al., 2021; Scheffers et al., 2017). This finding aligns with comorbidity patterns commonly observed in adolescence, whereby bullying co-occurs with body image disturbances, anxiety, depression, and PTSD, collectively contributing to a cross-symptom propagation mechanism (Dyer et al., 2012; Lee and Vaillancourt, 2018).

In addition, body appreciation emerged as a bridge between the symptom and victimization clusters, with an absolute BEI of 0.35. Notably, its signed BEI was −0.35, suggesting that body appreciation may serve as a protective bridging factor, mitigating rather than amplifying the linkage between victimization and symptomatology. A positive body image can predict self-esteem and emotional wellbeing (Linardon et al., 2023; Swami et al., 2018), thereby providing psychological resources for coping with bullying and PTSD symptoms. From a network intervention perspective, fostering body appreciation may reduce cross-community transmission from bullying to symptoms, representing an efficient strategy that targets bridging factors. Moreover, this underscores the role of positive psychological resources in trauma healing: rather than focusing exclusively on risk-related bridging factors, strengthening protective factors can likewise attenuate the activation of symptom networks.

In summary, alienation, as a bridging symptom within the PTSD cluster, exhibited the strongest cross-community connectivity, suggesting that it may constitute a key pathway through which external risks (e.g., bullying or body image concerns) are transmitted into the PTSD symptom network. Verbal bullying, by contrast, may represent a high-risk factor for such transmission between PTSD and body image disturbances. Finally, body appreciation, identified as a negative BEI node, appears to exert a psychological buffering effect.

### 4.2 Physical activity as a developmental protective factor

Extending these findings, the present study further examined the moderating role of physical activity in the interplay between bullying-related trauma risk factors, variables related to body image, and trauma symptoms. Moderated network analysis revealed that, even after accounting for physical activity, body appreciation exhibited a negative association with alienation (social isolation, negative self-appraisal, negative affect and pessimism about the future). Higher levels of body acceptance and appreciation are inversely associated with emotional detachment, self-devaluation, and negative affect in the context of bullying-related trauma. This finding is consistent with previous research, suggesting that positive body image may enhance self-esteem and psychological security, thereby fostering emotional connectedness and psychological stability (Courtwright et al., 2020). Analyses further indicated that physical activity moderates this relationship. Specifically, among individuals with low levels of physical activity, the negative association between body appreciation and alienation was stronger, whereas this relationship was attenuated among individuals with high levels of physical activity.

Leisure-time physical activities, especially sport, which involve enjoyment, teamwork and achievement (Berg et al., 2015), contrast sharply with alienation, such as social isolation, negative self-appraisal, negative affect and pessimism about the future. Typically, many forms of physical activity are accompanied by social opportunities (such as football, basketball and table tennis races, the most common sports among Chinese children). Within such contexts, the social interactions embedded in these activities may be intrinsically conducive to mitigating experiences of loneliness and alienation. As studies have found, leisure-time physical activity is an important tool for creating opportunities for social interactions and developing a sense of community (Grieve and Sherry, 2012; Warner et al., 2012). The formation of a sense of community is rooted in the fundamental human need to create and maintain social bonds, experience a sense of belonging, and develop a sense of self-identity (Skinner et al., 2008).

In addition, studies have shown that regular participation in physical activity also underpins self-esteem and emotion regulation (Giles et al., 2017; Ligeza et al., 2019), thereby enabling children to cope more adaptively with negative self-evaluations and adverse emotional experiences (Zhu and Zhang, 2026). The moderating role of physical activity should likewise be interpreted from a developmental perspective. Childhood represents a critical period for acquiring emotional regulation skills, developing peer relationships, and constructing self-identity. Leisure-time physical activity may therefore provide opportunities that are particularly valuable for children, not only by promoting physical health but also by facilitating social competence, emotional regulation, and adaptive coping.

Consequently, in contexts of higher physical activity, the associated positive outcomes, such as positive emotional experience, achievement, and a sense of social belonging, may constitute a form of compensatory mechanism. Thus, even when children appraise their bodies unfavorably, they may nonetheless derive psychological fulfillment through alternative pathways. Physical activity may serve as a source of psychological compensation and social capital, which in turn could mitigate the adverse effects of low body appreciation on alienation. However, a decline in physical activity participation may result in the loss of a related compensatory pathway, thereby exacerbating the relationship between low body appreciation and alienation.

Taken together, these findings indicate that participation in physical activity may play a buffering role in the relationship between body image and alienation. Among individuals with low exercise levels, positive body image functions as an important psychological resource for alleviating post-traumatic alienation. In contrast, among individuals with high exercise levels, the psychological and social benefits of exercise itself may, to some extent, substitute for the role of body image, thereby weakening their negative association.

### 4.3 Physical activity: protective and risk-related processes in body appreciation and verbal bullying

In addition, the study found that the negative relationship between body appreciation and verbal bullying was moderated by physical activity level. Specifically, no significant association was observed in the low-activity group, whereas a significant negative correlation emerged in the high-activity group. However, while the estimated pairwise associations (edge weight) were relatively stable, the three-way interaction underlying this moderation effect showed relatively wide confidence intervals and in the MNM analysis; therefore, the moderation effect should be interpreted with greater caution.

On the one hand, the negative association between body appreciation and verbal bullying can be interpreted within the frameworks of positive body image and self-concept theories. Body appreciation reflects an accepting and respectful attitude toward one’s body and has been consistently associated with higher self-esteem and greater psychological resilience (Tylka and Wood-Barcalow, 2015b; Tort-Nasarre et al., 2023). Individuals with higher levels of body appreciation are less likely to internalize negative appearance-related evaluations and are better able to maintain a stable self-concept in the face of verbal bullying, thereby attenuating its impact (Courtwright et al., 2020).

Regular engagement in physical activity has been found to be associated with enhanced body awareness and more positive body image (Sabiston et al., 2019; Srismith et al., 2023). In this context, individuals with higher levels of physical activity may be more likely to derive self-esteem from body-related evaluations and attitudes, which can strengthen the protective function of body appreciation. In addition, participation in physical activity is often accompanied by increased opportunities for social interaction and support (Grieve and Sherry, 2012), which may further consolidate positive body-related self-perceptions. As a result, the negative association between body appreciation and verbal bullying may be more pronounced among individuals with higher levels of physical activity.

On the other hand, individuals with higher levels of physical activity may place greater emphasis on bodily attributes in their self-worth and are more frequently exposed to contexts involving social comparison and evaluation (e.g., performance, physique, and appearance). Research has shown that individuals who engage in regular physical activity report higher self-esteem on appearance- and body-related dimensions compared with their less active counterparts, suggesting a stronger valuation of bodily attributes (Bobbio, 2009). Moreover, individuals with higher activity levels tend to develop a stronger exercise self-identity and are more frequently involved in social comparison processes related to athletic performance, physique, and appearance (Verkooijen and de Bruijn, 2013). Collectively, these findings suggest that higher levels of physical activity may increase the salience of bodily evaluation and the likelihood of social comparison. In such contexts, exposure to body-related negative comments may be more likely to be internalized, potentially undermining body image.

Body appreciation functions as a crucial psychological resource that buffers individuals from the adverse effects of bullying on mental health (Courtwright et al., 2020; Tylka and Wood-Barcalow, 2015b). Beyond its protective capacity, it also serves as a form of socialized self-expression, an avenue through which individuals negotiate identity and belonging within a social world. Physical activity, in this sense, becomes more than a health-promoting behaviour. It operates as a medium for the embodied articulation of self-identity, offering opportunities to reclaim agency and reconstruct a positive bodily narrative after experiences of social exclusion. Through engagement in leisure-time physical activity, individuals reassert their presence in the social space, transforming the body from a site of stigma into one of expression, connection, and self-recognition. Nevertheless, physical activity may also reinforce appearance-focused ideals in certain contexts, reminding us that its social meanings can be both liberating and constraining.

Therefore, the moderating role of physical activity in the relationship between verbal bullying and body image may be understood as reflecting a coexistence of protective and risk-related processes. On the one hand, physical activity may enhance positive body image, which in turn exerts a protective effect by fostering self-esteem and strengthening social support; on the other hand, it may also increase the salience of bodily evaluation, thereby potentially intensifying the adverse impact of bullying experiences on body image.

### 4.4 Clinical implications

The present findings have several practical implications for the prevention and intervention of bullying-related PTSD symptoms in children. First, the identification of alienation and verbal bullying as key bridging variables suggests that reducing social isolation and addressing verbal victimization may interrupt the transmission of psychological distress across symptom domains. Second, body appreciation emerged as a protective bridge factor, indicating that interventions aimed at fostering positive body image may help buffer the psychological impact of bullying. Finally, given the moderating role of physical activity, promoting regular leisure-time physical activity in accordance with WHO recommendations may provide an accessible and low-cost strategy to support emotional wellbeing, strengthen social connectedness, and enhance resilience among children exposed to bullying.

## 5 Limitation

Several limitations should be acknowledged when interpreting the present findings. First, although the moderation stability analysis with 1,000 bootstrap resamples has indicated acceptable robustness, the cross-sectional design precludes causal inferences regarding the directionality of associations among bullying victimization, body image variables, and PTSD symptoms. Future research can therefore employ randomized controlled trials in combination with MNM to examine the effects of physical activity interventions and to elucidate how these interventions influence the temporal dynamics embedded in the network. Second, all measures relied on child self-reports, which may be subject to recall bias, social desirability, and subjective misperceptions. Incorporating multi-informant perspectives (e.g., parents, teachers, or peers) alongside objective behavioral assessments would enhance the validity of future studies.

Finally, the sample was drawn from a specific cultural and educational context, which may limit the generalizability of the findings. Cross-cultural replications are warranted to determine whether the identified bridging variables and moderating mechanisms are consistent across diverse populations. In addition, the present study assessed overall leisure-time physical activity without distinguishing between specific subtypes, such as planned exercise and sport. Although both are commonly conceptualized within the broader domain of leisure-time physical activity, they may involve distinct behavioral characteristics and underlying mechanisms (e.g., physiological versus psychosocial pathways). Future research using more fine-grained measures is needed to disentangle the potentially differential effects of various forms of physical activity.

## 6 Conclusion

This study demonstrated that verbal bullying, alienation, and body appreciation served as critical bridging variables linking bullying victimization, PTSD symptoms and body image in children. Alienation emerged as a central pathway through which school bullying victimization and body image impact PTSD, while verbal bullying represented a high-risk factor connecting these domains. Importantly, body appreciation functioned as a protective factor, buffering the activation of risk-symptom networks.

Furthermore, leisure-time physical activity may mitigate the link between self-identity or self-appraisal, particularly regarding the body, and alienation by providing social and psychological compensatory resources, while potentially amplifying vulnerability to body-related stressors through social comparison. However, the moderation effects showed limited stability and therefore should be interpreted with caution.

## Data Availability

The original contributions presented in the study are included in the article/Supplementary material, further inquiries can be directed to the corresponding author.

## References

[ref1] American Psychiatric Association (2013). Diagnostic and Statistical Manual of mental Disorders. 5th Edn Arlington, VA: American Psychiatric Association.

[ref2] Ariani T. A. Putri A. R. Firdausi F. A. Aini N. (2025). Global prevalence and psychological impact of bullying among children and adolescents: a meta-analysis. J. Affect. Disord. 385:119446. doi: 10.1016/j.jad.2025.119446, 40393548

[ref3] Asmundson G. J. G. Frombach I. McQuaid J. Pedrelli P. Lenox R. Stein M. B. (2000). Dimensionality of posttraumatic stress symptoms: a confirmatory factor analysis of DSM-IV symptom clusters and other symptom models. Behav. Res. Ther. 38, 203–214. doi: 10.1016/S0005-7967(99)00061-3, 10661004

[ref4] Bear G. G. Gaskins C. Blank J. Chen F. F. (2011). Delaware school climate survey—student: its factor structure, concurrent validity, and reliability. J. Sch. Psychol. 49, 157–174. doi: 10.1016/j.jsp.2011.01.001, 21530762

[ref5] Bell V. Robinson B. Katona C. Fett A.-K. Shergill S. (2019). When trust is lost: the impact of interpersonal trauma on social interactions. Psychol. Med. 49, 1041–1046. doi: 10.1017/s0033291718001800, 30043717

[ref6] Berg B. K. Warner S. Das B. M. (2015). What about sport? A public health perspective on leisure-time physical activity. Sport Manag. Rev. 18, 20–31. doi: 10.1016/j.smr.2014.09.005

[ref7] Berne S. Frisén A. Kling J. (2014). Appearance-related cyberbullying: a qualitative investigation of characteristics, content, reasons, and effects. Body Image 11, 527–533. doi: 10.1016/j.bodyim.2014.08.006, 25194309

[ref8] Bobbio A. (2009). Relation of physical activity and self-esteem. Percept. Mot. Skills 108, 549–557. doi: 10.2466/pms.108.2.549-557, 19544960

[ref9] Brixval C. S. Rayce S. L. B. Rasmussen M. Holstein B. E. Due P. (2012). Overweight, body image and bullying--an epidemiological study of 11- to 15-years olds. Eur. J. Pub. Health 22, 126–130. doi: 10.1093/eurpub/ckr010 21382970

[ref10] Brown T. A. Cash T. F. Mikulka P. J. (1990). Attitudinal body-image assessment: factor analysis of the body-self relations questionnaire. J. Pers. Assess. 55, 135–144. doi: 10.1080/00223891.1990.9674053, 2231236

[ref11] Bull F. C. Al-Ansari S. S. Biddle S. Borodulin K. Buman M. P. Cardon G. . (2020). World Health Organization 2020 guidelines on physical activity and sedentary behaviour. Br. J. Sports Med. 54, 1451–1462. doi: 10.1136/bjsports-2020-102955, 33239350 PMC7719906

[ref12] Chetverikov D. Liao Z. Chernozhukov V. (2021). On cross-validated lasso in high dimensions. Ann. Stat. 49, 1300–1317. doi: 10.1214/20-aos2000

[ref13] Cloitre M. (2021). Complex PTSD: assessment and treatment. Eur. J. Psychotraumatol. 12:1866423. doi: 10.1080/20008198.2020.1866423 PMC8221157 34211640

[ref14] Courtwright S. E. Flynn Makic M. B. Jones J. (2020). Emotional wellbeing in youth: a concept analysis. Nurs. Forum 55, 106–117. doi: 10.1111/nuf.12404, 31677158

[ref16] Csikszentmihalyi M. (2009). Flow: The psychology of optimal experience. New York, NY: Harper & Row.

[ref17] Day S. Bussey K. Trompeter N. Mitchison D. (2021). The impact of teasing and bullying victimization on disordered eating and body image disturbance among adolescents: a systematic review. Trauma Violence Abuse 23, 985–1006. doi: 10.1177/1524838020985534, 33461439

[ref18] Deci E. L. Ryan R. M. (2008). Self-determination theory: a macrotheory of human motivation, development, and health. Can. Psychol. 49, 182–185. doi: 10.1037/a0012801

[ref19] Dyer A. Borgmann E. Kleindienst N. Feldmann R. E. Jr. Vocks S. Bohus M. (2012). Body image in patients with posttraumatic stress disorder after childhood sexual abuse and co-occurring eating disorder. Psychopathology 46, 186–191. doi: 10.1159/000341590, 22964627

[ref20] Edner B. J. Glaser B. A. Calhoun G. B. (2017). Predictive accuracy and factor structure of the child report of posttraumatic symptoms (CROPS) among adjudicated youth. Psychol. Trauma 9, 706–713. doi: 10.1037/tra0000303, 28682104

[ref21] Elhai J. D. Palmieri P. A. (2011). The factor structure of posttraumatic stress disorder: a literature update, critique of methodology, and agenda for future research. J. Anxiety Disord. 25, 849–854. doi: 10.1016/j.janxdis.2011.04.007, 21793239

[ref22] Epskamp S. Borsboom D. Fried E. I. (2018). Estimating psychological networks and their accuracy: a tutorial paper. Behav. Res. Methods 50, 195–212. doi: 10.3758/s13428-017-0862-1, 28342071 PMC5809547

[ref23] Fernández-Aldana M. Miranda R. Agámez P. Rodríguez-Posada A. Ferro E. (2024). Bullying and posttraumatic stress disorder (PTSD) symptoms among adolescents of public schools in Colombia: a cross-sectional study. J. Aggress. Maltreatment Trauma 33, 1025–1043. doi: 10.1080/10926771.2024.2331692

[ref24] Fox C. L. Farrow C. V. (2009). Global and physical self-esteem and body dissatisfaction as mediators of the relationship between weight status and being a victim of bullying. J. Adolesc. 32, 1287–1301. doi: 10.1016/j.adolescence.2008.12.006, 19157531

[ref25] Fraser-Thomas J. L. Côté J. Deakin J. (2005). Youth sport programs: an avenue to foster positive youth development. Phys. Educ. Sport Pedagog. 10, 19–40. doi: 10.1080/1740898042000334890

[ref26] Giles G. E. Cantelon J. A. Eddy M. D. Brunyé T. T. Urry H. L. Mahoney C. R. . (2017). Habitual exercise is associated with cognitive control and cognitive reappraisal success. Exp. Brain Res. 235, 3785–3797. doi: 10.1007/s00221-017-5098-x, 28975416

[ref27] Greenwald R. Rubin A. (1999). Assessment of posttraumatic symptoms in children: development and preliminary validation of parent and child scales. Res. Soc. Work. Pract. 9, 61–75. doi: 10.1177/104973159900900105

[ref28] Grieve J. Sherry E. (2012). Community benefits of major sport facilities: the Darebin international sports Centre. Sport Manag. Rev. 15, 218–229. doi: 10.1016/j.smr.2011.03.001

[ref29] Haslbeck J. M. B. (2022). Estimating group differences in network models using moderation analysis. Behav. Res. Methods 54, 522–540. doi: 10.3758/s13428-021-01637-y, 34291432 PMC8863727

[ref30] Haslbeck J. M. B. Borsboom D. Waldorp L. J. (2021). Moderated network models. Multivar. Behav. Res. 56, 256–287. doi: 10.1080/00273171.2019.1677207, 31782672

[ref31] Haslbeck J. M. B. Waldorp L. J. (2018). How well do network models predict observations? On the importance of predictability in network models. Behav. Res. Methods 50, 853–861. doi: 10.3758/s13428-017-0910-x, 28718088 PMC5880858

[ref32] Holubcikova J. Kolarcik P. Madarasova Geckova A. Van Dijk J. P. Reijneveld S. A. (2015). Is subjective perception of negative body image among adolescents associated with bullying? Eur. J. Pediatr. 174, 1035–1041. doi: 10.1007/s00431-015-2507-7, 25708851

[ref33] Idsoe T. Dyregrov A. Idsoe E. C. (2012). Bullying and PTSD symptoms. J. Abnorm. Child Psychol. 40, 901–911. doi: 10.1007/s10802-012-9620-0, 22391775

[ref34] Idsoe T. Vaillancourt T. Dyregrov A. Hagen K. A. Ogden T. Nærde A. (2020). Bullying victimization and trauma. Front. Psych. 11:480353. doi: 10.3389/fpsyt.2020.480353, 33519533 PMC7841334

[ref35] Janoff-Bulman R. (1992). Shattered Assumptions: Towards a new Psychology of Trauma. New York, NY: Free Press.

[ref36] Jansen W. S. Otten S. van der Zee K. I. Jans L. (2014). Inclusion: conceptualization and measurement. Eur. J. Soc. Psychol. 44, 370–385. doi: 10.1002/ejsp.2011

[ref37] Jones P. J. Ma R. McNally R. J. (2021). Bridge centrality: a network approach to understanding comorbidity. Multivar. Behav. Res. 56, 353–367. doi: 10.1080/00273171.2019.1614898, 31179765

[ref38] Kaess M. (2018). Bullying: peer-to-peer maltreatment with severe consequences for child and adolescent mental health. Eur. Child Adolesc. Psychiatry 27, 945–947. doi: 10.1007/s00787-018-1201-5, 30003397

[ref39] Lee K. S. Vaillancourt T. (2018). Longitudinal associations among bullying by peers, disordered eating behavior, and symptoms of depression during adolescence. JAMA Psychiatry 75, 605–612. doi: 10.1001/jamapsychiatry.2018.0284, 29641816 PMC6137525

[ref40] Li T. Chen B. Li Q. Wu X. Li Y. Zhen R. (2023). Association between bullying victimization and post-traumatic stress disorders among Chinese adolescents: a multiple mediation model. BMC Psychiatry 23:758. doi: 10.1186/s12888-023-05212-x, 37848816 PMC10580599

[ref41] Liang D. (1994). College students’ stress levels and their relationship to physical exercise. Chin. J. Ment. Health 8, 5–6.

[ref42] Liao X. M. (2007). The Evaluation of the Childhood and Adolescence Trauma Events Questionnaire and the Report of Post-Traumatic Symptoms [Master’s Thesis]. Guangzhou: Jinan University.

[ref43] Ligeza T. S. Kałamała P. Tarnawczyk O. Maciejczyk M. Wyczesany M. (2019). Frequent physical exercise is associated with better ability to regulate negative emotions in adult women: the electrophysiological evidence. Ment. Health Phys. Act. 17:100294. doi: 10.1016/j.mhpa.2019.100294

[ref44] Linardon J. Anderson C. McClure Z. (2023). Body appreciation predicts better mental health and wellbeing. A short-term prospective study. Body Image 45, 20–24. doi: 10.1016/j.bodyim.2023.02.001, 36764235

[ref45] Linardon J. McClure Z. Tylka T. L. Fuller-Tyszkiewicz M. (2022). Body appreciation and its psychological correlates: a systematic review and meta-analysis. Body Image 42, 287–296. doi: 10.1016/j.bodyim.2022.07.003, 35878528

[ref46] Liu H. Jia S. Wang J. (2022). Yoga leisure and identity development of Chinese second-time mothers. J. Leis. Res. 53, 56–74. doi: 10.1080/00222216.2020.1864238

[ref47] Liu H. Petukhova M. V. Sampson N. A. Aguilar-Gaxiola S. Alonso J. Andrade L. H. . (2017). Association of DSM-IV posttraumatic stress disorder with traumatic experience type and history in the World Health Organization world mental health surveys. JAMA Psychiatry 74, 270–281. doi: 10.1001/jamapsychiatry.2016.3783 28055082 PMC5441566

[ref48] Ma R. (2006). Initial Revision of MBSRQ and the Research on Relativity Betwteen MBSRQ and Personality types. [Master’s thesis]. Xi’an, China: Fourth Military Medical University.

[ref49] Midei A. J. Matthews K. A. (2011). Interpersonal violence in childhood as a risk factor for obesity: a systematic review of the literature and proposed pathways. Obes. Rev. 12, e159–e172. doi: 10.1111/j.1467-789X.2010.00823.x, 21401850 PMC3104728

[ref50] Mikkelsen E. G. E. Einarsen S. (2002). Basic assumptions and symptoms of post-traumatic stress among victims of bullying at work. Eur. J. Work Organ. Psychol. 11, 87–111. doi: 10.1080/13594320143000861

[ref51] Nielsen M. B. Tangen T. Idsoe T. Matthiesen S. B. Magerøy N. (2015). Post-traumatic stress disorder as a consequence of bullying at work and at school. A literature review and meta-analysis. Aggress. Violent Behav. 21, 17–24. doi: 10.1016/j.avb.2015.01.001

[ref52] Olweus D. (2013). School bullying: development and some important challenges. Annu. Rev. Clin. Psychol. 9, 751–780. doi: 10.1146/annurev-clinpsy-050212-185516, 23297789

[ref53] Ossa F. C. Pietrowsky R. Bering R. Kaess M. (2019). Symptoms of posttraumatic stress disorder among targets of school bullying. Child Adolesc. Psychiatry Ment. Health 13:43. doi: 10.1186/s13034-019-0304-1, 31728159 PMC6842197

[ref54] Pearce M. J. Boergers J. Prinstein M. J. (2002). Adolescent obesity, overt and relational peer victimization, and romantic relationships. Obes. Res. 10, 386–393. doi: 10.1038/oby.2002.53, 12006638

[ref55] Pieper J. (2009). Leisure: the Basis of Culture. San Francisco: Ignatius Press.

[ref56] Poling D. V. Smith S. W. Taylor G. G. Worth M. R. (2019). Direct verbal aggression in school settings: a review of the literature. Aggress. Violent Behav. 46, 127–139. doi: 10.1016/j.avb.2019.01.010

[ref57] Raskauskas J. Rubiano S. Offen I. Wayland A. K. (2015). Do social self-efficacy and self-esteem moderate the relationship between peer victimization and academic performance? Soc. Psychol. Educ. 18, 297–314. doi: 10.1007/s11218-015-9292-z

[ref58] Rosenbaum S. Vancampfort D. Steel Z. Newby J. Ward P. B. Stubbs B. (2015). Physical activity in the treatment of post-traumatic stress disorder: a systematic review and meta-analysis. Psychiatry Res. 230, 130–136. doi: 10.1016/j.psychres.2015.10.017, 26500072

[ref59] Sabiston C. M. Pila E. Vani M. Thogersen-Ntoumani C. (2019). Body image, physical activity, and sport: a scoping review. Psychol. Sport Exerc. 42, 48–57. doi: 10.1016/j.psychsport.2018.12.010

[ref60] Scheffers M. van Busschbach J. T. Bosscher R. J. Aerts L. C. Wiersma D. Schoevers R. A. (2017). Body image in patients with mental disorders: characteristics, associations with diagnosis and treatment outcome. Compr. Psychiatry 74, 53–60. doi: 10.1016/j.comppsych.2017.01.004, 28095340

[ref61] Sentenac M. Arnaud C. Gavin A. Molcho M. Gabhainn S. N. Godeau E. (2012). Peer victimization among school-aged children with chronic conditions. Epidemiol. Rev. 34, 120–128. doi: 10.1093/epirev/mxr024, 22130095

[ref62] Skinner J. Zakus D. H. Cowell J. (2008). Development through sport: building social capital in disadvantaged communities. Sport Manag. Rev. 11, 253–275. doi: 10.1016/S1441-3523(08)70112-8

[ref63] Smith P. K. (2012). Cyberbullying: challenges and opportunities for a research program—a response to Olweus (2012). Eur. J. Dev. Psychol. 9, 553–558. doi: 10.1080/17405629.2012.689821

[ref64] Spence Laschinger H. K. Nosko A. (2015). Exposure to workplace bullying and post-traumatic stress disorder symptomology: the role of protective psychological resources. J. Nurs. Manag. 23, 252–262. doi: 10.1111/jonm.12122, 24033807

[ref65] Srismith D. Dierkes K. Zipfel S. Thiel A. Sudeck G. Giel K. E. . (2023). Physical activity improves body image of sedentary adults. Exploring the roles of interoception and affective response. Curr. Psychol. 42, 26663–26671. doi: 10.1007/s12144-022-03641-7

[ref66] Stebbins R. A. (2012). The Idea of Leisure: First Principles. New Brunswick, NJ: Transaction Publishers.

[ref67] Stebbins R. A. (2015). “The serious leisure perspective,” in Leisure and Positive Psychology: Linking Activities with Positiveness, ed. Stebbins R. A. (London: Palgrave Macmillan UK), 11–40.

[ref68] Swami V. Weis L. Barron D. Furnham A. (2018). Positive body image is positively associated with hedonic (emotional) and eudaimonic (psychological and social) well-being in British adults. J. Soc. Psychol. 158, 541–552. doi: 10.1080/00224545.2017.1392278, 29053404

[ref69] Thijs J. Verkuyten M. (2008). Peer victimization and academic achievement in a multiethnic sample: the role of perceived academic self-efficacy. J. Educ. Psychol. 100, 754–764. doi: 10.1037/a0013155

[ref70] Tort-Nasarre G. Pollina-Pocallet M. Ferrer Suquet Y. Ortega Bravo M. Vilafranca Cartagena M. Artigues-Barberà E. (2023). Positive body image: a qualitative study on the successful experiences of adolescents, teachers and parents. Int. J. Qual. Stud. Health Well Being 18:2170007. doi: 10.1080/17482631.2023.2170007, 36710436 PMC9888451

[ref71] Tylka T. L. Wood-Barcalow N. L. (2015a). The body appreciation Scale-2: item refinement and psychometric evaluation. Body Image 12, 53–67. doi: 10.1016/j.bodyim.2014.09.006, 25462882

[ref72] Tylka T. L. Wood-Barcalow N. L. (2015b). What is and what is not positive body image? Conceptual foundations and construct definition. Body Image 14, 118–129. doi: 10.1016/j.bodyim.2015.04.001, 25921657

[ref73] Verkooijen K. T. de Bruijn G.-J. (2013). Exercise self-identity: interactions with social comparison and exercise behaviour. Psychol. Health Med. 18, 490–499. doi: 10.1080/13548506.2012.750727, 23323686

[ref74] Wang J. Iannotti R. J. Nansel T. R. (2009). School bullying among adolescents in the United States: physical, verbal, relational, and cyber. J. Adolesc. Health 45, 368–375. doi: 10.1016/j.jadohealth.2009.03.021, 19766941 PMC2751860

[ref75] Warner S. Dixon M. A. Chalip L. (2012). The impact of formal versus informal sport: mapping the differences in sense of community. J. Community Psychol. 40, 983–1003. doi: 10.1002/jcop.21506

[ref76] Wei Y. Xie J. Zhu Z. (2019). Patterns of bullying victimization among adolescents in China: based on a latent profile analysis. Best Evid. Chin. Educ. 3, 361–375. doi: 10.15354/bece.19.ar1270

[ref77] Wheaton B. (2010). Understanding Lifestyle Sports: Consumption, Identity and Difference [1 publ., transferred to digital printing]. London: Routledge.

[ref78] Wu W. Li D. Zhou H. Wang K. Tylka T. L. (2024). Psychometric properties of a mandarin Chinese version of the body appreciation Scale-2 among Chinese adolescents. Body Image 50:101739. doi: 10.1016/j.bodyim.2024.101739, 38820800

[ref79] Xie J. Lv Y. Bear G. Yang C. Marshall S. Gong R. (2015). The validity and reliability of the Chinese version of Delaware bullying victimization scale – student. Chin. J. Clin. Psychol. 23, 594–596. doi: 10.16128/j.cnki.1005-3611.2015.04.006

[ref80] Xie J. Wei y. Bear G. (2018). Revision of Chinese version of Delaware bullying victimization scale-student in adolescents. Chin. J. Clin. Psychol. 26, 259–263.

[ref81] Zhao N. Yang S. Zhang Q. Wang J. Xie W. Tan Y. . (2024). School bullying results in poor psychological conditions: evidence from a survey of 95,545 subjects. Front. Psychol. 15:1279872. doi: 10.3389/fpsyg.2024.1279872, 38328372 PMC10847557

[ref82] Zhu H. Carr E. Carr N. (2026). From mundane to everyday leisure: rethinking happiness, meanings, and life state through multispecies’ narrative. Leis. Sci., 48:1–18. doi: 10.1080/01490400.2026.2643249

[ref83] Zhu H. Zhang Y. (2025). The ‘attachment style-leisure’ framework: a conceptualized framework for transformation between stressful exercises and leisure activities. Leis. Sci., 1–18. doi: 10.1080/01490400.2025.2568046, 37339054

[ref9001] Zhu H. Zhang Y. (2026). The moving brain: A cross-pathways framework linking exercise to the modulation of aversive information processing. Mental Health and Physical Activity, 30:100757. doi: 10.1016/j.mhpa.2026.100757, 37339054

