# Supplementary material for: Exploring how leisure-time physical activity moderate the school bullying–trauma network among children: the role of body image
Source: Front Psychol. 2026 Aug 19;17:1804968. doi: 10.3389/fpsyg.2026.1804968 (PMC13533647; doi:10.3389/fpsyg.2026.1804968)
Supplement: Supplementary file 2 [file Presentation_2.pdf]

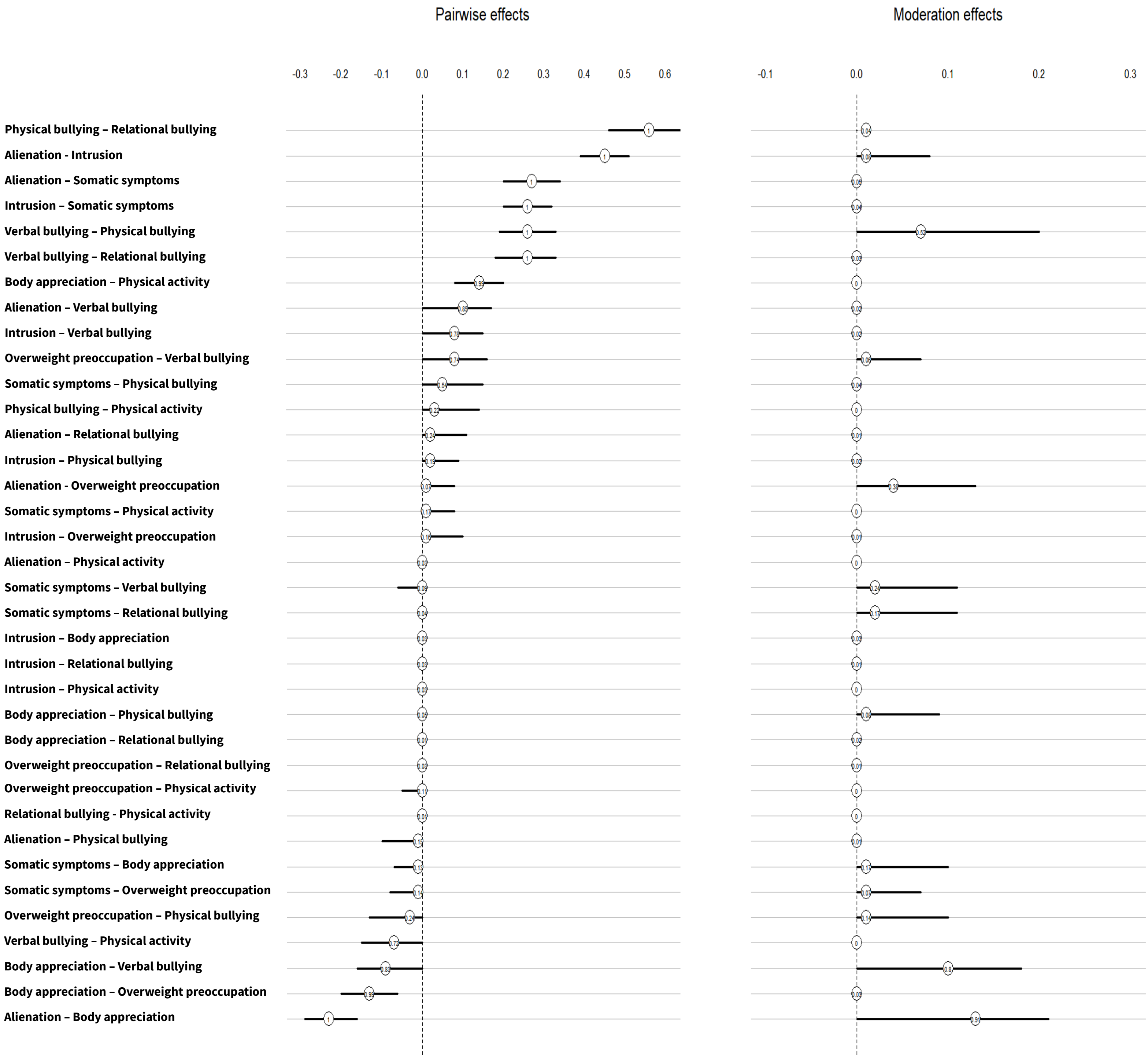

Fig S2. The nonparametric bootstrapped sampling distributions of the MNM
