# Supplementary material for: Exploring how leisure-time physical activity moderate the school bullying–trauma network among children: the role of body image
Source: Front Psychol. 2026 Aug 19;17:1804968. doi: 10.3389/fpsyg.2026.1804968 (PMC13533647; doi:10.3389/fpsyg.2026.1804968)
Supplement: Supplementary file 3 [file Presentation_3.pdf]

Supplementary Materials: Psychometric Properties of Chinese Versions of the Measures

Delaware Bullying Victimization Scale–Student Form (Chinese DBVS-S)
The total scale showed excellent internal consistency with a Cronbach's α of 0.906 and good test–retest reliability over a three-week interval, with a reliability coefficient of 0.783. The subscales also demonstrated acceptable internal consistency, with verbal bullying α = 0.776, physical bullying α = 0.763, and relational bullying α = 0.799 (Xie, Wei, & Bear, 2018).

Child Report of Post-Traumatic Symptoms (Chinese CROPS)
The scale showed high internal consistency with a Cronbach's α of 0.903 and satisfactory test–retest reliability over a five-week period, with a reliability coefficient of 0.765 (Liao, 2007).

Body Appreciation Scale-2 (Chinese BAS-2)
The Mandarin Chinese version of the BAS-2 demonstrated strong internal consistency with McDonald's ω of 0.92. The test–retest reliability over a three-week interval was 0.63, indicating moderate stability (Wu, Li, Zhou, Wang, & Tylka, 2024).

Overweight Preoccupation Subscale of the Multidimensional Body–Self Relations Questionnaire (Chinese MBSRQ)
This subscale showed acceptable internal consistency with a Cronbach's α of 0.752. The test–retest reliability over a four-week interval was 0.65, suggesting moderate temporal stability (Ma, 2006).

Xie, J., Wei, y., & Bear, G. (2018). Revision of Chinese version of Delaware bullying victimization scale-student in adolescents. *Chinese Journal of Clinical Psychology, 26*(2), 259-263.

Liao, X. M. (2007). *The evaluation of the Childhood and Adolescence Trauma Events Questionnaire and the Report of Post-Traumatic Symptoms* [Master's thesis, Jinan University].

Wu, W., Li, D., Zhou, H., Wang, K., & Tylka, T. L. (2024). Psychometric properties of a

Mandarin Chinese version of the Body Appreciation Scale-2 among Chinese adolescents. *Body Image, 50*, 101739. doi:https://doi.org/10.1016/j.bodyim.2024.101739

Ma, R. (2006). Initial Revision of MBSRQ and the Research on Relativity betwteen MBSRQ and personality types. *Fourth Military Med Univ*.
